# Sociodemographic and reproductive risk factors associated with obesity in a population of brazilian women from the city of Ribeirão Preto: a cross-sectional study

**DOI:** 10.1186/s12889-023-16056-1

**Published:** 2023-06-23

**Authors:** Ênio Luis Damaso, Heloisa Bettiol, Viviane Cunha Cardoso, Carolina Sales Vieira, Elaine Christine Dantas Moisés, Ricardo Carvalho Cavalli

**Affiliations:** 1 Department of Gynecology and Obstetrics, Ribeirao Preto Medical School, University of Sao Paulo, Ribeirao Preto, Sao Paulo, Brazil; 2 Department of Pediatrics, Ribeirao Preto Medical School, University of Sao Paulo, Ribeirao Preto, Sao Paulo, Brazil

**Keywords:** Obesity, Pregnancy, Cohort study, Predictive factors

## Abstract

**Background:**

Obesity is a highly prevalent chronic disease that is associated with the development of other metabolic comorbidities. Its etiology is complex and multiple risk factors have been reported. In women, weight gain during pregnancy and the effect of pregnancy on subsequent weight gain are important events in women’s history. Both pregnancy and postpartum are critical periods for the development of obesity.

**Objectives:**

To identify sociodemographic and reproductive risk factors associated with obesity in women in their fourth decade of life.

**Methods:**

Cohort study conducted on women born from June 1978 to May 1979 in Ribeirão Preto, Brazil. Sociodemographic, clinical, and obstetric data were collected by interview and clinical evaluation. Univariable and multivariable binomial logistic regression models were constructed to identify the risk factors of obesity and the adjusted relative risk (RR) was calculated.

**Results:**

The cohort included 916 women and 309 (33.7%) of them were obese. Obesity was associated with low educational level (RR 1.77, 95%CI 1.33–2.35) and teenage pregnancy (RR 1.46, 95%CI 1.10–1.93). There was no association of obesity with the other covariates studied.

**Conclusion:**

Obesity is associated with years of schooling and teenage pregnancy.

## Introduction

Obesity is a chronic disease defined by the World Health Organization (WHO) as abnormal or excessive fat accumulation that presents a risk to health [1]. In adults, overweight is defined as a body mass index (BMI) 25 to 29.9 kg/m^2^ and obesity as BMI ≥ 30 kg/m^2^ [1, 2].

It has been increasing in epidemic proportions in both adults and children [3, 4]. The global prevalence of obesity has increased by 50%, from 8.7% to 2000 to 13.1% in 2016 [4]. In Brazil, the prevalence of overweight and obesity among adults was 55.4% and 20.3% in 2019, respectively, showing an increase of 72% over the last 13 years [5].

Many chronic conditions that reduce longevity and quality of life are caused or affected by obesity, including diabetes mellitus, systemic arterial hypertension, dyslipidemia, metabolic syndrome (association of these previous conditions), and cardiovascular diseases [6, 7]. The burden of these diseases is extremely high among lower-income countries and populations. A total of 63% (36 million) of global death occurred in 2008 due to lifestyle diseases or non-communicable diseases (NCDs) [7].

Different scholars mention a lot of predisposing factors which vary depending on geography, social conditions, political and economic factors, and human genetics. In aggregate, the commonest factors were sociodemographic, behavioral, genetic, and living in obesogenic environment. Many of the risk factors are known; however, a major gap in the scientific literature is how these factors are interrelated [8]. Knowing more about the interaction between these factors may be the key to developing better treatment and prevention measures.

There are multiple factors and processes that lead to obesity. In fact, the traditional view is usually that the main cause is the excess energy stored in fat cells than the energy the body used. In addition, more and more etiologies or defects that lead to obesity can be identified under the lights of social, genetic, epigenetic, environmental and microenvironment issues [9].

While there has been a plethora of articles about obesity published worldwide [1, 2, 7, 8] and covering Brasil as a unique region [5, 10], there are a lot of lacks have not been published yet. As the Brazil country is very huge it is important to focus on smaller geographical parts and subjects as they have different level of development and the predictors might play diverse roles in the prevalence of obesity. Epidemiological knowledge permits to design public policies for the prevention and promotion of healthy habits in a society. Primary prevention efforts are especially important in obesity management since studies have shown that, once obesity is established, it is difficult to reverse [11] and will continue into adulthood [12].

Women tend to be more overweight and obese than males around the world and studies have shown that some NCDs have a predilection for women [13]. This work seeks to study obese in adult women and to answer the question whether pregnancy is a risk factor for overweight and obesity. Going through the pregnancy process would be a risk factor for accumulating fat and becoming obese.

The primary objective of the present study was to identify sociodemographic and reproductive risk factors associated with obesity in women in their fourth decade of life, from a birth cohort that has been analyzed since 1978/79. The secondary objectives were to identify reproductive risk factors of obesity in women with previous pregnancies, as well as to describe the frequency of obesity and other chronic diseases (diabetes mellitus, systemic arterial hypertension, dyslipidemia, and metabolic syndrome) in women in their fourth decade of life.

## Materials and methods

This is an analytical, observational cross-sectional study (nested in a cohort study). The sample of the present study consisted of women from the 1978/79 birth cohort conducted in the city of Ribeirão Preto, State of São Paulo, Brazil [14]. This is the first Brazilian cohort that evaluated these data.

Ribeirão Preto is a city in the State of São Paulo, a rich and industrialized region with a Human Development Index (HDI) of 0.800 in 2010. Its population was 604,682 inhabitants in 2010 [15]. It is one of the most developed cities in the country where 99% of residences have running water and are equipped with a sewage system [15].

**Fig. 1:**
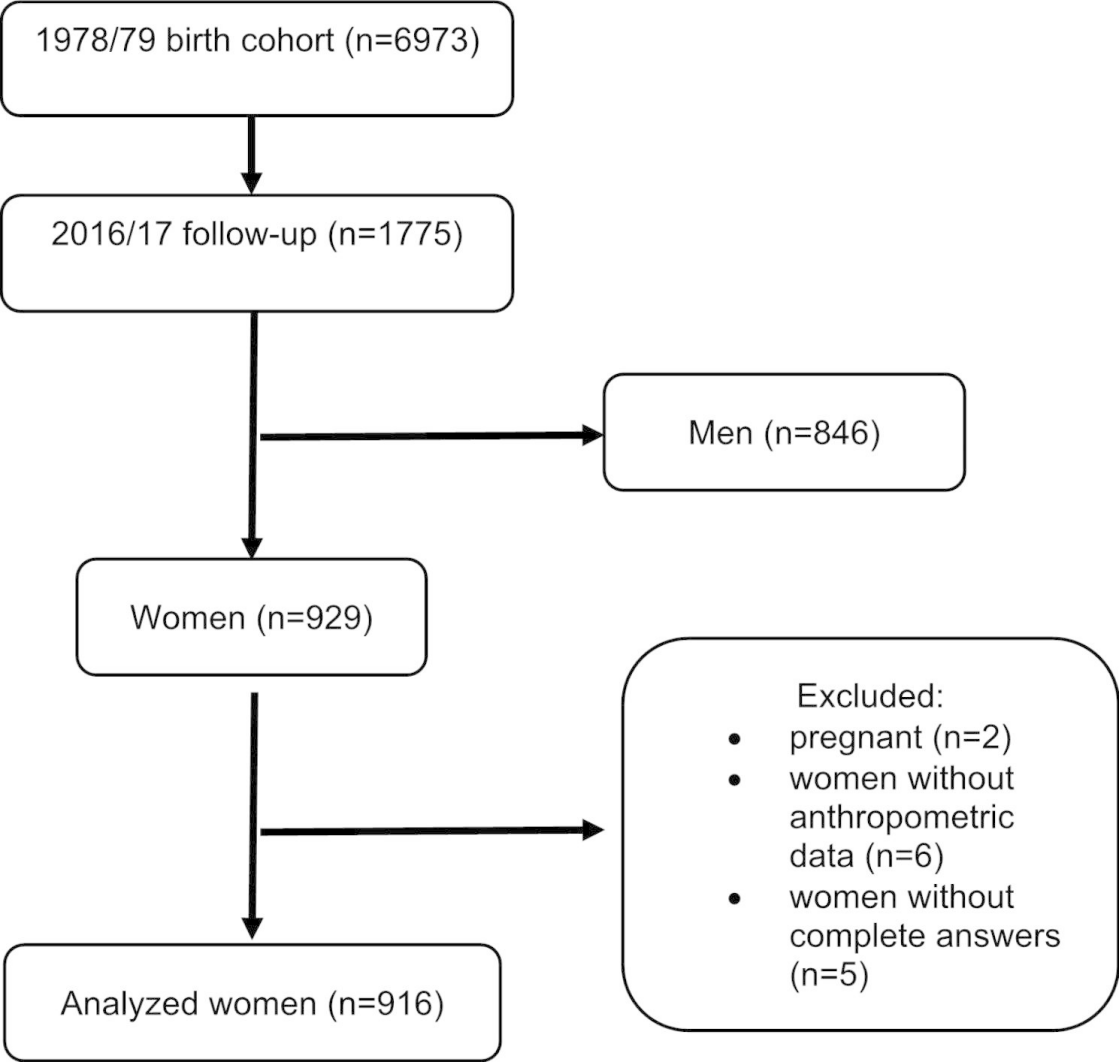
Flow diagram of participants in the 1978/79 Ribeirão Preto birth cohort

For the cohort study, the records and charts of the eight maternity hospitals (three public and five private) that attended 98% of all deliveries in the municipality [14] from June 1978 to May 1979 (n = 6,973) were reviewed [16]. During the following two years, civil registry offices were visited in the city to monitor deaths that had occurred in the first year of life of the children born during the period [14]. The first follow-up of this cohort occurred in 1987/1989, when the children were sought in schools [17]; 2,898 children aged 8 to 11 years were evaluated [18]. In 1996/1997, 2,083 male participants aged 18 or 19 years were evaluated on the occasion of enlistment in military service [17]. Between 2002 and 2004, the cohort was again visited and 2,103 participants aged 23 to 25 years were assessed [14]. The last follow-up of this cohort occurred in 2016/2017 when 1,775 participants (25% of the initial sample) aged 37 to 39 years were evaluated (Fig. 1).

This research has been performed in accordance with the Declaration of Helsinki and has been approved by the Research Ethics Committee of the School of Medicine of Ribeirão Preto, University of São Paulo. All stages of this cohort were submitted to the Research Ethics Committee. Before the review of records and charts of the eight maternity hospitals in 1978/79, permission was obtained from all clinical directors and the registry offices. The interviewed mothers from whom data and data of their children were collected provided oral consent and were released by their responsible physicians [14]. The last follow-up of the study was submitted to the Research Ethics Committee of the University Hospital, Ribeirão Preto Medical School, University of São Paulo (FMRP-USP), and was approved under number 1.282.710.

Women who participated in the 2016/17 assessment were included in the present study (n = 929). The participants were invited to come to the research center on a scheduled day and time. Eligible participants received information about the objectives of the study and were invited to sign the free informed consent form. Data collection was started only after the participants had signed the form.

Women who were pregnant at the time of assessment (n = 2), women who did not undergo collection of the anthropometric data (n = 6), and women who did not answer the questionnaire about obstetric history (n = 5) were excluded. Pregnant women were excluded because the BMI cut of 30 kg/m^2^ may not reflect the diagnosis of obesity depending on the gestational age.

Questionnaires including demographic, social, clinical, and reproductive data were applied during the interviews. Sociodemographic and reproductive health-related variables were included in this study. The following sociodemographic variables were collected: race/ethnicity (white and others), paid work (to be engaged in a paid activity at the time of application of the questionnaire), socioeconomic class, stable marital status (to live with a partner [married, cohabiting or stable relationship] or not [single, widowed]), educational level (years of schooling: ≤ 8 years, 9–11 years, ≥ 12 years), smoking (it was considered present if the patient answered that she had the habit of smoking, regardless of the number of cigarettes), alcohol misuse (alcohol abuse was considered to be the consumption of more than 3 doses in a day or more than 7 doses in a week, each dose being the equivalent of 10 g of alcohol [19]), and illicit drug use (the habit of using any illicit drug [cocaine, marijuana, opiates, volatile solvents and hallucinogens] was marked as present., regardless of the frequency of use). The sexual and reproductive health-related variables included previous pregnancy, number of previous pregnancies (0, 1 e ≥ 2), age at first pregnancy (< 20 years, 20 a 29 years, > 30 yaers), previous abortion, parity (0, 1 e ≥ 2), previous cesarean section, and breastfeeding (breastfeeding more than half of the children or less).

Socioeconomic status was defined according to the ABEP categories [20]. The estimated monthly household income for each ABEP category was: A (> 20× minimum wage), B (10–20× minimum wage), C (4–10× minimum wage), D (2–4× minimum wage), and E (< 2× minimum wage) [20]. The minimum wage in Brazil at completion of this study was US$267.81 per month.

The participants underwent body composition assessment, anthropometry, blood collection, and blood pressure measurement. Trained health professionals collected the data. Information on chronic diseases was obtained by interview. An existing condition was defined when the participant had a diagnosis or was being treated for systemic arterial hypertension, diabetes mellitus, and dyslipidemia. The criteria defined by the National Cholesterol Education Program Adult Treatment Panel III (NCEP-ATP III), revised by the American Heart Association and the National Heart, Lung and Blood Institute (AHA/NHLBI) [21], were considered for the diagnosis of metabolic syndrome.

Height was measured using a stadiometer graduated in centimeters and fixed to a smooth wall, with the patient standing erect, barefoot, with arms extended along the body, head positioned in the Frankfurt plane (imaginary line from the external auditory canal to the orbit lower eye) with eyes fixed on a point at eye level, legs parallel and heels, calves, buttocks, shoulder blades and the back of the head (occipital region) against the stadiometer or wall. Weight was measured using a Welmy ® digital anthropometric scale, with a capacity of 200 kg and accuracy of 100 g. The patient was positioned in the center of the scale, barefoot, erect, arms extended along the body, with feet together and in such a way that the weight was distributed symmetrically, avoiding the support more firmly on one of the legs and with the gaze fixed. at a point on the straight line.

Based on measured weight and height measurements, BMI was calculated according to the formula BMI = weight (kg)/height (m^2^) and classified according to the World Health Organization [1] as underweight (≤ 18.5 kg/m2), normal weight (between 18.6 and 24.9 kg/m2), overweight (between 25 and 29.9 kg/m2) and obese (≥ 30 kg/m2). The different BMI categories were grouped for better analysis, initially BMI ≤ 24.9 kg/m2, BMI between 25 and 29.9 kg/m2 and BMI ≥ 30 kg/m2. And for analysis of the outcome of the study, obese individuals (BMI ≥ 30 kg/m2) and non-obese (BMI ≤ 29.9 kg/m2) were considered. Obesity was the primary outcome variable of the [1]. For the analysis of risk factors, the women were divided into two groups: an obese group and a non-obese group.

To look for factors associated with obesity, univariate analysis was conducted using the covariates described above. All variables with *P* < 0.10 in the univariate analysis were included in a multiple logistic regression model.

After analysis of the factors associated with obesity in all women of the study, patients who had never been pregnant were excluded. The aim was to analyze the reproductive and obstetric characteristics associated with obesity in women with a previous pregnancy. The same statistical strategy was used for this analysis, obtaining the unadjusted and adjusted RR by binomial logistic regression. All variables with P < 0.10 were included in the multivariate model.

The data of each patient were entered into Excel spreadsheets for the creation of a database. The SAS 9.3 program (SAS Institute Inc., Cary, NC, USA) was used for all statistical analyses. A level of significance of 5% (p < 0.05) was adopted. Missing data were excluded from the analysis.

## Results

The stage used in this study had 1759 individuals who attended for data collection. Of these individuals, those of the male gender were excluded (n = 846), allowing the analysis of a total of 929 women. Women who were pregnant at the time of assessment (n = 2), women who did not undergo collection of the anthropometric data (n = 6), and women who did not answer the questionnaire about obstetric history (n = 5) were excluded. Then, 916 women were analyzed.

Most of them (80.2%) were white, had paid work (90%), lived with a companion (67.2%), had more than 12 years of schooling (45.9%), belonged to socioeconomic class A/B (68%), had a previous pregnancy (77.7%), were non-smokers (88.8%), were not abusive alcohol drinkers (78.4%), and did not use illicit drugs (96.6%) (Table 1).

**Table 1:** Sociodemographic and clinical characteristics of women in the 1978/79 Ribeirão Preto cohort (n = 916)

Variable		n	%
Race/ethnicity	White	735	80.2
	Other (black, brown, yellow)	181	19.8
Paid work	Yes	720	90.0
	No	80	10.0
Marital status	With a companion	614	67.2
	Without a companion	300	32.8
Educational level (years of schooling)	≤ 8	110	12.1
	9–11	382	42.0
	≥ 12	417	45.9
Socioeconomic class	A/B	598	68.0
	C	262	29.7
	D/E	20	2.3
Previous pregnancy	Yes	712	77.7
	No	202	22.3
BMI classification	Low weight or adequate	289	31.6
	Overweight	318	34.7
	Obesity	309	33.7
Diabetes mellitus	Yes	102	11.2
	No	811	88.8
Dyslipidemia	Yes	167	18.3
	No	744	81.7
Arterial hypertension	Yes	178	19.5
	No	734	80.5
Metabolic syndrome	Yes	286	31.2
	No	630	68.8
Smoking	Yes	102	11.2
	No	812	88.8
Alcohol misuse	Yes	171	21.6
	No	620	78.4
Illicit drug use	Yes	31	3.4
	No	385	96.6

Legend: n, number; %, percentage; BMI, body mass index

The prevalence of overweight (BMI between 25 and 29.9 kg/m^2^) and obesity was 34.7% and 33.7%, respectively. The prevalence of clinical comorbidities was 11.2% for diabetes mellitus, 19.5% for systemic arterial hypertension, 18.3% for dyslipidemia, and 31.2% for metabolic syndrome (Table 1).

Table 2 shows the binomial logistic multiple regression analysis performed to identify predictors of obesity. After multivariate analysis, less than 8 years of schooling remained as the only demographic factor associated with obesity (RR 1.77, 95% CI 1.33–2.35) when compared to more than 12 years of schooling (educational level). The other covariates were not associated with obesity. There was no collinearity between the variables included in the multiple regression models.

**Table 2:** Sociodemographic and reproductive factors associated with obesity in women of the 1978/79 Ribeirão Preto cohort (n = 916)

	Obesity (BMI ≥ 30 kg/m^2^)					
Variable	Yes (n = 309)	No (n = 607)	Unadjusted RR^a^ (95% CI)	Adjusted RR^b^ (95% CI)		
	n (%)	n (%)				
Race/ethnicity						
White	242 (78.3)	493 (81.2)	1.00 (reference)			
Other	67 (21.7)	114 (18.8)	1.12 (0.90–1.40)			
Paid work						
Yes	243 (89)	477 (90.5)	1.00 (reference)			
No	30 (11)	50 (9.5)	1.11 (0.82–1.50)			
Marital status						
With a partner	216 (70.1)	398 (65.7)	1.00 (reference)			
Without a partner	92 (29.9)	208 (34.3)	1.14 (0.93–1.40)			
Educational level						
**≤ 8 years**	**56 (18.4)**	**54 (8.9)**	**1.79 (1.41–2.28)**	**1.77 (1.33–2.35)**		
9–11 years	131 (42.9)	251 (41.6)	1.21 (0.98–1.48)	1.21 (0.97–1.50)		
≥ 12 years	118 (38.7)	299 (49.5)	1.00 (reference)	1.00 (reference)		
Socioeconomic class						
A/B	191 (64.3)	407 (69.8)	1.00 (reference)	1.00 (reference)		
C	101 (34.0)	161 (27.6)	1.20 (0.99–1.46)	1.00 (0.81–1.24)		
D/E	5 (1.7)	15 (2.6)	0.78 (0.36–1.68)	0.54 (0.25–1.19)		
Previous pregnancy						
Yes	249 (80.8)	461 (76.1)	1.21 (0.95–1.53)			
No	59 (19.1)	145 (23.9)	1.00 (reference)			
Number of pregnancies						
0	59 (19.1)	145 (23.9)	1.00 (reference)	1.00 (reference)		
1	76 (24.7)	172 (28.4)	1.05 (0.79–1.40)	1.05 (0.78–1.41)		
≥ 2	173 (56.2)	289 (47.7)	1.29 (1.01–1.65)	1.16 (0.89–1.51)		
Smoking						
Yes	36 (11.7)	66 (10.9)	1.05 (0.79–1.39)			
No	272 (88.3)	540 (89.2)	1.00 (reference)			
Alcohol misuse						
Yes	62 (24.5)	109 (20.2)	1.17 (0.93–1.48)			
No	191 (75.5)	429 (79.7)	1.00 (reference)			
Illicit drug use						
Yes	13 (4.2)	18 (3.0)	1.25 (0.82–1.91)			
No	296 (95.8)	589 (97.0)	1.00 (reference)			

^a^ Simple binomial logistic regression

^b^ Multiple binomial logistic regression

Legend: BMI, body mass index; n, number; %, percentage; RR, relative risk; 95% CI, 95% confidence interval

Variables included in the multivariate model: educational level, socioeconomic class, and number of pregnancies

After analysis of the factors associated with obesity in all women of the study, patients who had never been pregnant were excluded. After multivariate analysis, only teenage pregnancy (before 20 years of age) continued to be associated with obesity in women with a previous pregnancy when compared to age at first pregnancy over 30 years (RR 1.46, 95% CI 1.10–1.93). The other covariates were not associated with obesity. Table 3 shows the unadjusted and adjusted RR of the covariates studied. There was no collinearity between the variables included in the multiple regression models.

**Table 3:** Reproductive and obstetric factors associated with obesity in women of the 1978/79 Ribeirão Preto cohort who had at least one pregnancy (n=712)

	Obesity (BMI ≥ 30 kg/m^2^)					
Variable	Yes (n = 250)	No (n = 462)	Unadjusted RR^a^ (95% CI)	Adjusted RR^b^ (95% CI)		
	n (%)	n (%)				
Age at first pregnancy						
< 20 years	89 (37.3)	115 (26.3)	1.46 (1.10–1.93)	1.46 (1.10–1.93)		
20–29 years	100 (41.8)	205 (46.8)	1.10 (0.83–1.46)	1.10 (0.83–1.46)		
≥ 30 years	50 (20.9)	118 (26.9)	1.00 (reference)	1.00 (reference)		
Previous abortion						
Yes	60 (24)	124 (26.8)	1.00 (reference)			
No	190 (76)	338 (73.2)	1.10 (0.87–1.39)			
Parity						
0	11 (4.4)	23 (5.0)	1.00 (reference)			
1	79 (31.6)	198 (42.8)	0.88 (0.52–1.48)			
≥ 2	160 (64)	241 (52.1)	1.23 (0.74–2.03)			
Previous cesarean section						
Yes	166 (66.4)	314 (68)	0.95 (0.77–1.17)			
No	84 (33.6)	148 (32)	1.00 (reference)			
Breastfeeding (> 50%)						
Yes	217 (86.8)	404 (87.4)	1.00 (reference)			
No	33 (13.2)	58 (12.6)	1.03 (0.77–1.39)	1.11 (0.79–1.57)		

^a^ Simple binomial logistic regression

^b^ Multiple binomial logistic regression

Legend: BMI, body mass index; n, number; %, percentage; RR, relative risk; 95% CI, 95% confidence interval

Variables included in the multivariate model: age at first pregnancy and breastfeeding

## Discussion

The population consisted of women aged 37 to 39 years from a cohort study conducted in the city of Ribeirão Preto. Participants were recruited by telephone, advertisements through media and on social networks, and searches in a digital environment, strategies that may have been unable to reach marginalized groups. Thus, only 1,775 of 6,973 women were available for data collection. This sample corresponds to 25% of the initial sample, which means a loss of 75% of individuals. This loss can bring an important bias to the results of this study.

A significant proportion of the population was overweight (68.4%) and approximately one-third was obese (33.7%). Although different methods were used for data collection, these percentages are much higher than the Brazilian estimates obtained in the same year (2016) by the VIGITEL survey (Surveillance System for Risk and Protective Factors for Chronic Diseases by Telephone Survey), in which the prevalence of overweight was 53.7%, with 17.7% of obese adults and 19% of obese women aged 35 to 44 years [22].

The high prevalence of obesity found here agrees with North American estimates, which were slightly higher than the Brazilian rates. Data from the National Health and Nutrition Examination Survey (NHANES) showed a prevalence of obesity of 40% among adults aged 20 to 39 years in the United States; when stratified by age, there were 40.3% of obese men in this age group and 39.7% of obese women [23]. The high rates of overweight and obesity in the population studied indicate that the sample is vulnerable to other metabolic disorders and consequently to an increased cardiovascular risk [6].

The difference between the national prevalence of obesity and the prevalence found in this study may be due to some limitations of this cohort follow-up, including the recruitment process, the number of women from the initial cohort who attended the data collection, and the fact that the data were from a single city.

This percentage of women who were not followed may have brought some bias to the study, since it may determine a more frequent profile in the study. For example, women with more comorbidities may be more interested in health studies than women without chronic diseases, which would increase the prevalence of obesity in the sample.

The characteristics of the women participating in the 2016/17 assessment rendered the population more homogeneous. The majority of these women were white, had more than 8 years of schooling, lived with a companion, had paid work, and belonged to higher socioeconomic classes; these conditions may also have brought some bias to the study. The fact that the study gathered a more homogeneous and biased population, perhaps due to the greater demand for participation in the study by women with chronic diseases, such as obesity, the primary outcome of the study, may have hindered the statistical analysis. For example, this difficulty may have limited the association of obesity with previous pregnancy. Besides, the question that the data were from a single Brazilian city can limit the generalizability of the present findings.

The women were divided into two groups according to the presence or absence of obesity to identify factors associated with this disorder. The only associated sociodemographic factor was a low educational level (less than 8 years of schooling compared to more than 12 years) and the only reproductive health-related factor was teenage pregnancy (< 20 years). In the case of women with higher educational level, possible explanations are greater social pressure and better access to weight control and weight loss programs, regardless of whether or not they are healthy [24, 25]. On the other hand, women with low educational level may have difficulty understanding better food choices and have limited access to weight loss programs. In teenage mothers, both sociodemographic and physiological risk factors for obesity are present. The sociodemographic risk factors include black race/ethnicity, poverty, and low educational level [26, 27]. Physiological risk factors are higher gestational weight gain and greater postpartum weight retention than that observed in adults [28].

In contrast to expectations, this study did not find an association between other reproductive factors and obesity. Pregnancy and postpartum are critical periods for the development of obesity; however, although the relationship between maternal pregnancy weight and the risk of becoming obese has been the focus of studies in recent years [29, 30], the level of evidence is still dubious. Studies like the present one rely on the physiology of gestational weight gain in part at the expense of body fat accumulation during pregnancy in an attempt to show the association between previous pregnancies and obesity [31, 32].

Several Brazilian studies aimed to estimate the prevalence of obesity and to identify associated factors. One study reported a prevalence of obesity of 16.8% among men and of 24.4% among women in 2013/2014. Advanced age (over 50 years), low educational level (no schooling or incomplete elementary school), African descent, and living with a partner were risk factors for obesity. Leisure-time physical activity and the habit of watching more than 4 h of television per day had significant effects in both sexes. Regarding morbidity, obese people were more likely to have a diagnosis of hypertension, diabetes, or non-communicable chronic diseases [33].

A study conducted in 2011 in Rio de Janeiro revealed a higher prevalence of abdominal obesity (waist circumference > 80 cm) among women older than 35 years with two or more children. When the analyses were stratified by BMI category, parity and age were no longer significantly associated with abdominal obesity [34]. Another study conducted in 2006 in São Leopoldo, Rio Grande do Sul, found an inverse association between educational level and obesity, with a higher prevalence of overweight and obesity among women with a lower educational level. Women belonging to the lowest quartile were 33% more likely to be overweight than women with 11 or more years of schooling, a finding that can be explained in part by poor living conditions [35]. In 2011, a study conducted in Criciúma, Santa Catarina, showed that more than 60% of adult women had some degree of overweight or obesity and that 44% had central obesity. Most of the reproductive factors studied were not associated with obesity; however, women with three or more children were more likely to be overweight or obese [36].

One major strength of this study is its cohort design. Birth cohort studies have been a top priority on the research and technology agenda of developed countries [37]. The assessment of a group of live births over a given period allows to monitor the health of these individuals throughout their lives [37].

In summary, the present results are in line with the global scenario of obesity being a highly prevalent disease in adults. In women, obesity is associated with a low educational level and teenage pregnancy. Primary prevention strategies are necessary, and attention must be paid to women with low educational level and to pregnant adolescents. During prenatal care of teenagers, interventions that promote appropriate weight gain are vital to prevent postpartum weight retention because excess gestational weight gain is a strong predictor of maternal overweight and obesity after pregnancy [27].

## Data Availability

The data and materials that support the findings of this study are available from the corresponding author, Ênio Luis Damaso, upon reasonable request.
